# Morphology and Nomenclature of *Barsassia* (Lycopsida) from the Middle Devonian of West Junggar, Xinjiang, China

**DOI:** 10.3390/plants10122631

**Published:** 2021-11-30

**Authors:** Bingcai Liu, Kai Wang, Ruiwen Zong, Yi Wang, Honghe Xu

**Affiliations:** 1 State Key Laboratory of Palaeobiology and Stratigraphy, Nanjing Institute of Geology and Palaeontology and Center for Excellence in Life and Paleoenvironment, Chinese Academy of Sciences, Nanjing 210008, China; bcliu@nigpas.ac.cn (B.L.); kaiwang@nigpas.ac.cn (K.W.); yiwang@nigpas.ac.cn (Y.W.); 2 University of Chinese Academy of Sciences, Beijing 100049, China; 3 State Key Laboratory of Biogeology and Environmental Geology, China University of Geosciences, Wuhan 430074, China; zong_1658@126.com

**Keywords:** nomenclature, *Barsassia*, Devonian, paleobotany, Xinjiang

## Abstract

Morphology and nomenclature are essential issues of botany, in which both extant and fossil plant taxa follow the same nomenclature code. Devonian (419.2–358.9 Ma) herbaceous lycopsid *Barsassia*, one of the earliest coal-forming plants in geological history, possesses a characteristic, easily recognized, step-like stem and has been thought to be an index fossil for dating and correlating the Middle Devonian strata, especially those in the paleoblocks of Siberia, Kazakhstan, Xinjiang, and North China. Here, we systematically study the Devonian lycopsid *Barsassia* in terms of its morphology and nomenclature, based on the new materials from the Middle Devonian Hujiersite Formation of West Junggar, Xinjiang, China, and the International Code of Nomenclature for algae, fungi, and plants (Shenzhen Code). *Barsassia ornata* is determined as the type species of the genus, and a neotype is designated for that name. *Barsassia ornata* consists of fan- or rectangular-shaped leaves with awl-shaped or finger-like distal tips. Its leaves are pseudo-whorls and imbricately arranged on the stem surface forming distinct step-like structure.

## 1. Introduction

The Devonian Period (419.2–358.9 million years ago) is critical for the origin and the radiation of terrestrial vascular plants. All land plant groups except angiosperm appeared in this period [1], as well as with the rise of the first forest [2,3] that brought profound impacts on Earth’s environment and ecosystem, e.g., a dramatic drop of atmosphere CO_2_ and the extensive terrestrial weathering [4] caused by plants rooting system development [5,6]. Devonian plant fossil records show a critical and distinctive window for understanding the plant and paleoenvironment evolution [7].

The herbaceous lycopsid *Barsassia* has been thought to be an important fossil taxon for dating and correlating the Middle Devonian strata, especially those in Siberia, Kazakhstan, Xinjiang, and North China paleoblocks [8,9,10,11,12,13], for its characteristic and easily recognized step-like stem. Much terrestrial organic carbon produced from Devonian *Barsassia* and other lycopsids was buried and formed the earliest coal seams [14] and potential petroleum reserve, e.g., only 80–100 cm thick carbonaceous beds in the Middle Devonian of Junggar, Xinjiang [12,13,15,16], Kuznetsk Basin, southwestern Siberia [17,18], and Luquan, Yunnan, southern China [14,19].

*Barsassia* was established by Zalessky in 1933 [17] based on materials from the Middle Devonian of Barsas, Siberia, Russia. There have been four species of *Barsassia* since then, including the type species *Barsassia ornata* from the Middle Devonian of Siberia and North Xinjiang [17] characterized by fleshy fan-shaped leaf, *Barsassia platyphylla* from the Middle Devonian of Siberia, Russia [20], *Barsassia sibirica* from the Middle Devonian of Junggar, Xinjiang, China [9], *Barsassia grandis* with rectangular-shaped leaf from the Middle Devonian of Kazakhstan [21], and *Barsassia plana* with broad fan-shaped leaf from the Middle Devonian of Kazakhstan [21]. However, the morphological characteristics of different species of *Barsassia* and their nomenclature are controversial, as a result of little attention paid to Central Asian materials and the Russian literature.

In this study, we recognize the diagnostic characters of the genus *Barsassia* based on materials from the Middle Devonian Hujiersite Formation of Junggar, Xinjiang, and we fulfill the names of the genus and its type species *Barsassia ornata* and designate the neotype for the species according to the International Code of Nomenclature for algae, fungi, and plants (Shenzhen Code) [22].

## 2. Materials and Methods

All plant fossils were collected from the Hongguleleng Section (GPS: 46°39′32″ N, 85°1′34″ E) and the Gannaren Section (GPS: 46°37′25″ N, 86°4′23″ E), Hoxtolgay, Hoboksar County, West Junggar Basin, Xinjiang Uygur Autonomous Region, China (see Figure 1 of [13]). Specimens are preserved as compression, impression, and cast in coal seam. The coal seam is a mark layer of the Upper Member of the Hujiersite Formation [12,13]. Abundant plant fossils were reported from the Hujiersite Formation, including *Haskinsia hastata*, *Haskinsia sagittata* [23], *Leclercqia uncinata* [24], *Colpodexylon gracilentum* [25], *Hoxtolgaya robusta* [26], *Drepanophycus minor* [27], and *Aneurophyton doui* [28]. The geological age of Hujiersite Formation was determined as Middle-Late Devonian (Givetian to early Frasnian) based on evidence from study of palynology [15], megaplant fossils [12,15], and radioisotope [29].

Specimens studied here are highly carbonized so that it is impossible to obtain any biological molecular information but only the morphology of our fossil plant. Some fossil rock samples were treated using standard palynological method, namely the HF-HCL-HF acid maceration procedure, including processing the samples in 30% HCl, washing in distilled water to neutral, demineralization in 60% HF with repeated stirring, and mounting organic matters from the residues before sieving using 15 μm mesh. A few significant spores were obtained from the fossil samples but not illustrated in this study.

Fossil plants are partially covered by rock matrix, and complete morphological features can’t be observed directly from materials just collecting from the field. We removed the rock matrix using sharp tungsten needles under microscope to reveal fossil plant morphology features (dégagement: [30]). Macrophotography was carried out using a Nikon D800E digital camera with 105 mm macro-lens and Leica M205C microscope. Morphological measurements were obtained using the ImageJ software. All illustrated specimens are housed at the Nanjing Institute of Geology and Palaeontology, Chinese Academy of Sciences, with reference numbers and the prefix PB.

## 3. Systematic Paleobotany

Class: Lycopsida

Order: Drepanophycales

Family: Asteroxylaceae Kidston et Lang, 1920 [31]

Genus: *Barsassia* (Zalessky, 1933) [17]

Type species: *Barsassia ornata* (Zalessky, 1933) [17]

Generic diagnosis: Herbaceous lycopsid. Stem bifurcated, surface with step-like structure formed by pseudo-whorls, tightly, and imbricately arranged leaves. Leaf persistent, fan-shaped or rectangular-shaped with a distal leaf tip. Stele star-shaped, tracheid spirally thickened.

Remark: The leafy stem of *Barsassia* conforms the characters of the Class Lycopsida. Snigirevskaya and Bogdanova (1992) [31] reported its star-shaped stele and assumed its anatomical similarities to *Asteroxylon* [32,33]. We here follow their classification and assign *Barsassia* to the Order Drepanophycales and the Family Asteroxylaceae.

Species: *Barsassia ornata* [17].

**Figure 1:**
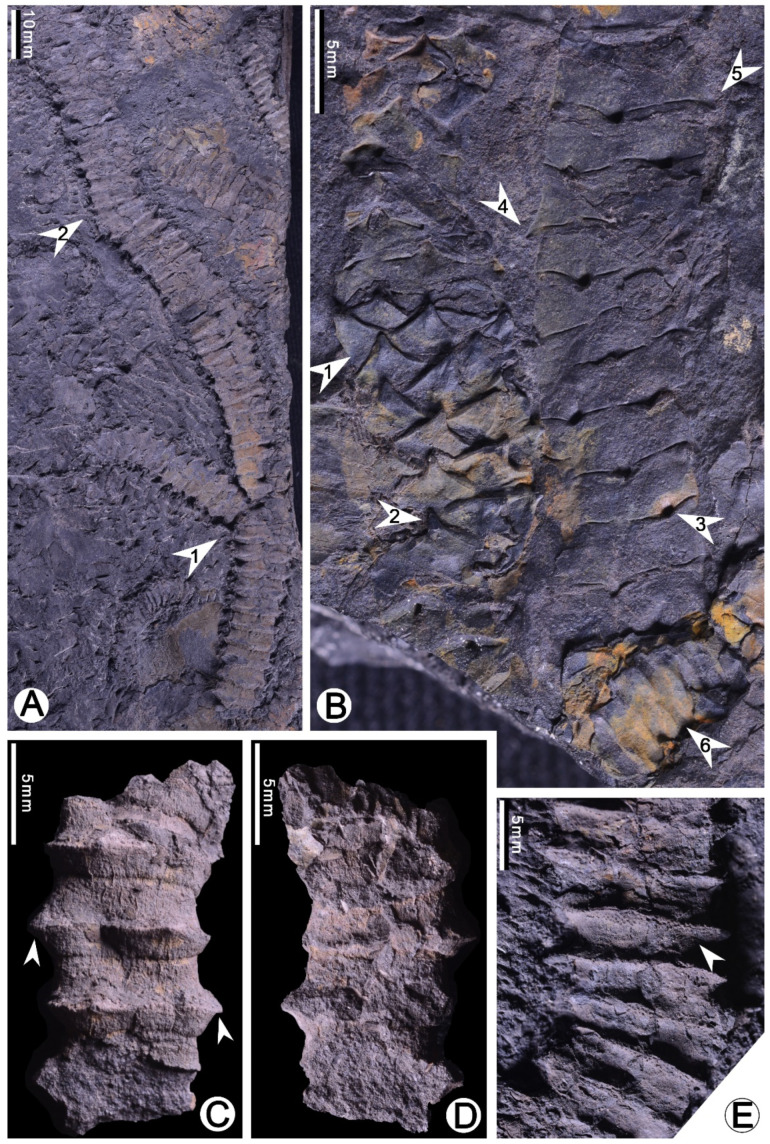
*Barsassia ornata* [17] from the Middle Devonian Hujiersite Formation, Hoxtolgay, West Junggar, Xinjiang, China. (**A**) A bifurcated leafy stem with obvious step-like structure on the surface. PB23702. (**B**) Neotype. Two stems with imbricately and tightly arranged leaves, leaf shape in a variety ranging from fan-shaped (arrow 1), fan-shaped with short awl-shaped tips (arrow 2), and rectangular-shaped (arrow 3) in surface view. Arrow 6 indicates the step-like stem. PB23703. (**C**,**D**) A three-dimensional cast of the stem with tipped leaves (arrows), one and its reverse side PB23704. (**E**) Enlargement of a portion of leafy stem indicated by arrow 2 in A, showing the step-like structure of the stem and dots on leaf surface (arrow).

**Figure 2:**
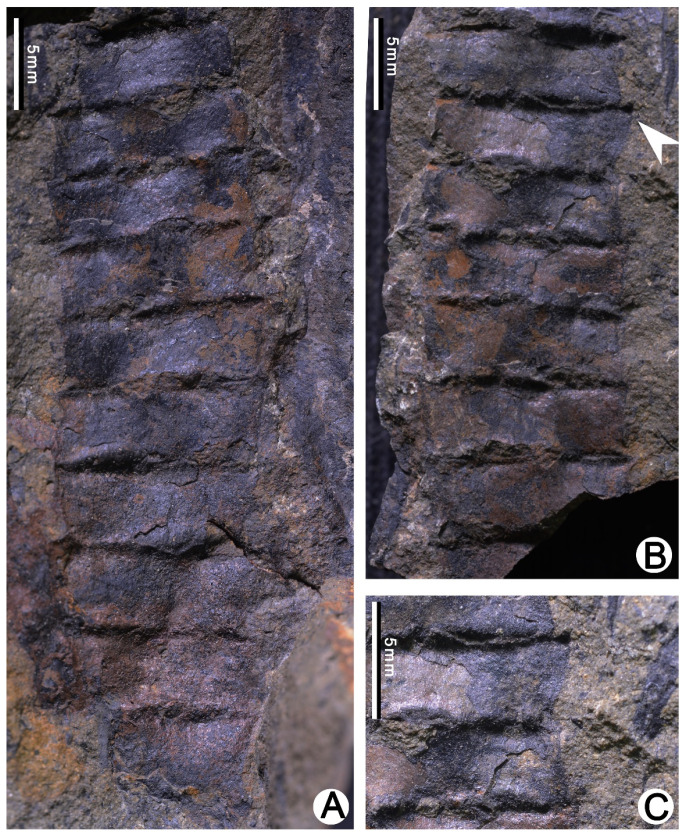
*Barsassia ornata* [17] from the Middle Devonian Hujiersite Formation of the Gannaren Section, West Junggar, Xinjiang, China. (**A**,**B**) Part and counterpart specimens showing the leafy stem with step-like structure and rectangular-shaped leaf with lateral tip (arrow). PB23705A, PB23705B. (**C**) Enlargement of the arrowed portion in B, showing the leaf in lateral view.

Synonyms (this synonym list shows that all illustrated specimens under below-listed names are transferred to *Barsassia ornata*, although some of these names are identical to ours): 

1933 *Barsassia ornata*, Zalessky, Figures 1 and 2.

**Figure 3:**
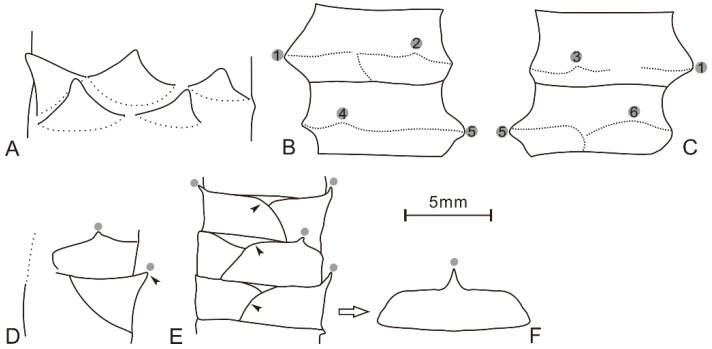
Line-drawings of *Barsassia ornata* [17] leafy stem based on materials from Middle Devonian Hujiersite Formation, West Junggar, Xinjiang, China. (**A**) From arrow 1 of Figure 1B, showing the fan-shaped leaves in surface view and the arrangement of leaves. (**B**,**C**) From Figure 1C,D. Dotted line indicates the leaf margin; the gray disks indicate leaf tips; and leaf tips are numbered and correspond in part and counterpart specimens. (**D**) From arrow 4 of Figure 1B, showing leaves in lateral view with tips (grey disks). (**E**). From arrowed portion of Figure 2B, showing the leaf in lateral view with obvious tip (gray disk) and the rectangular-shaped leaf. Black arrows show the leaf margin. The lateral leaf shows rectangular-shaped leaf after unfolding (**F**).

**Figure 4:**
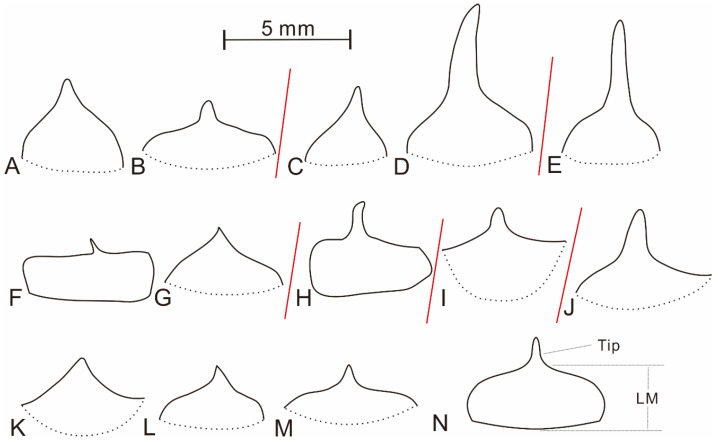
Line-drawings of leaf appearances of *Barsassia ornata* [17] from different localities and a leaf shape diagram. (**A**) From Figure 1 of [17], under the name of *Barsassia ornata* from the Middle Devonian of Barzas, southwestern Siberia, Russia. (**B**) From Figure 2 of [17], under the name of *Barsassia ornata* from the Middle Devonian of Barzas, southwestern Siberia, Russia. (**C**–**D**) From Plate XVV. Figure 8 of [20], under the name of *Barsassia ornata* from the Middle Devonian of Barzas, southwestern Siberia, Russia. (**E**) From Plate XVV. Figure 2 of [20], under the name of *Barsassia platyphylla* from the Middle Devonian of Barzas, southwestern Siberia, Russia. (**F**) From Plate 201. Figure 11 of [9]; under the name of *Barsassia sibirica* from the Middle Devonian of West Junggar Basin, Xinjiang, China. (**G**) From Plate 201. Figure 4 of [9], under the name of *Barsassia sibirica* from the Middle Devonian of West Junggar Basin, Xinjiang China. (**H**) From Plate LXIV. Figure 2 of [21], under the name of *Barsassia grandis* from the Middle Devonian of Katbas Mountains, Kazakhstan. (**I**): From Plate LXIV. Figure 6 of [21], under the name of *Barsassia plana* from the Middle Devonian of Katbas Mountains, Kazakhstan. (**J**) From Plate 4 of [34], under the name of *Barsassia ornata* from the Middle Devonian of southwestern Siberia, Russia. (**K**–**M**) Based on the Figure 1B of this study, under the name of *Barsassia ornata* from the Middle Devonian of West Junggar Basin, Xinjiang China. (**N**) A diagram of leaf shape showing the leaf main body (LM) and tip.

1975 *Barsassia ornata*, Stepanov, Plate XXV, Figures 1–6 and 9.

1975 *Barsassia platyphylla*, Stepanov, Plate XXV, Figures 7 and 8.

1983 *Barsassia sibirica*, Dou et al., Plate 201, Figures 3–5, 9, 11 and 12.

1983 *Barsassia sibirica*, Huang, Plate II, Figures 4–6.

1991 *Barsassia grandis*, Senkevitsch, Plate LXIV, Figures 1–4.

1991 *Barsassia plana*, Senkevitsch, Plate LXIV, Figures 5–7.

1992 *Barsassia ornata*, Snigirevskaya and Bogdanova, Plate III, Figures 1 and 2.

2010 *Barsassia ornata*, Snigirevskaya, Plates 1, 3–6.

2021 *Barsassia sibrica*, Liu et al., Figure 3A,B.

Specific diagnosis: Stem at least 161 mm long and 6.6–12 mm wide without counting leaves. Leaves tightly, pseudo-whorls, and imbricately arranged on the stem forming step-like structure, 3–6 leaves per gyre. Leaf with fan- or rectangular-shaped main body and a short awl-shaped to long finger-like distal tip. The distal tip ranges from not visible to up to 1.1 mm long. The whole leaf up to 1.7–4.4 mm in height and 3.3–4.5 mm in width.

Neotype: PB23703 (Figure 1B).

Horizon and Distribution: The Upper Member of the Hujiersite Formation (Givetian, Middle Devonian), Hoxtolgay, Hoboksar County, West Junggar Basin, Xinjiang Uygur Autonomous Region, China. Fuxingtun Formation (Middle Devonian), Zhangguangcai Mountains, Heilongjiang Province, northeastern China. Barzas Formation (Lower-Middle Devonian), Kuznetsk Basin, southwestern Siberia, Russia. Kazakh Horizon (Middle Devonian), Balkhash Land, Kazakhstan.

Description: The description is based on twenty specimens of our collection, from which 5 pieces are selected and illustrated here. All specimens are leafy stem and conform to the same plant morphology and belong to one plant. No fertile structure is discovered from the whole collection.

From all specimens, leaves are seen to be attached to the stem, indicating that the plant has persistent leaves. The stem is straight or slightly sinuous, at least 161 mm long, and dichotomously branched (Figure 1A), 6.6–12 mm (mean value = 10.3 mm, n = 13) wide without counting laterally attached leaves.

Leaves are tightly imbricate and pseudo-whorls arranged on the stem surface, forming step-like structure with points on the surface (Figure 1 and Figure 2). Three leaves are seen on the single surface in a 10 mm wide stem, indicating that the plant has six leaves per gyre (Figure 1B and Figure 3A). Leaf number per gyre is related to stem width, and in a 6.5 mm wide stem (Figure 1C,D), the number is three. From all specimens of our collection, we see 3–6 leaves per gyre.

The leaf is fan- or rectangular-shaped with a distal tip (Figure 1B–D, Figure 3A and Figure 4K–M). The surface of the specimens is undulate seen under microscope, and the leaf margin can be clearly observed (Figure 2 and Figure 3E). The leaf is entirely marginal, 1.7–4 mm (mean value = 2.9 mm, n = 13) in height, i.e., from the base of the leaf to the distal tip, 3.7–4.5 mm (mean value = 4.1 mm, n = 10) wide in its basal portion. The distal tip varies in appearance ranging from short awl-shaped to long finger-like shaped. In some cases, the tip is not visible and occasionally up to 1.1 mm in length. The leaf main body (LM), i.e., the part without counting the tip (Figure 4N), normally has nearly horizontal upper edges. As a result, LM usually shows rectangular shape in surface view (Figure 2 and Figure 3E). The rectangle formed by the LM is clearly edged and slightly fluctuated, and comprises one of the most easily recognized characters of the present plant. The single rectangle is measured as 2.4–4.4 mm (mean value = 3.4 mm, n = 12) in height (Figure 1B, Figure 2 and Figure 3B–F) and 3.3–4.5 mm (mean value = 3.8 mm, n = 7) in width. The leaf in lateral view shows the thick LM with typical upward tip (Figure 1B arrow 4, 5; Figure 1C,D and Figure 2B,C).

## 4. Discussion

Zalessky (1933) [17] firstly reported *Barsassia ornata* based on two specimens from the Middle Devonian of Barsas, Siberia. Zalessky (1933) [17] described the leaf of *Barsassia ornata* as isosceles triangular shape with a slightly pointed tip, and the leaf surface was covered with dots (Figures 1 and 2 of [17] and Figure 4A,B of this study). Actually, the plant’s leaf shape showed varied appearance not typical isosceles triangular shape even fan-shaped leaves are also seen from the original illustrations (Figures 1 and 2 of [17]). According to the International Code of Nomenclature for algae, fungi, and plants (Shenzhen Code), the publication of *Barsassia ornata* was effective (Articles 29–31 of [22]) for distributing the printed matter (through sale, exchange, or gift) to the general public. From 1 January 1912 to 1 January 1996 the valid publication of a fossil species must provide the illustrations or figures showing the essential character of the fossil-taxon (Article 43 of [22]), and did not require the type specimens before 1 January, 1958 (Article 40 of [22]). Since 1 January, 1958, the holotype designation has been obligatory to a valid publication of a fossil species (Article 40 of [22]). So, the original publication of *Barsassia ornata* in 1933 [17], though no holotype was designated nor illustrated, only two sketches of original specimens were given (Figures 1 and 2 of [17]), and the specimens storage keeps unknown, was valid but imperfect. According to the Article 7.7 of [22], the type is an element selected from the entire context of the validation description or diagnosis, unless the validating author designate a different type; therefore, the type of *Barsassia ornata* needs to be selected from Zalessky (1933)’s original protologue. However, the type of a fossil-taxon at the rank of species or below is always a specimen (Article 8.5 of [22]) rather than the original illustrations of Zalessky (1933) [17]. So, it is necessary to select neotype from the new materials. In order to fulfill the name of the species *Barsassia ornata*, we select the neotype from our materials from Xinjiang.

Stepanov (1975) [20] accepted the name *Barsassia ornata* and added new specific characters of leaf with a narrow awl-shaped leaf tip and expanded base (Plate XVV, Figures 1–6 and 9 of [20] and Figure 4C,D of this study) based on materials from the Middle Devonian of southwestern Siberia, Russia. Additionally, Stepanov (1975) [20] established the second species, *Barsassia platyphylla*, which was characterized by triangular leaf with a wide base and an elongated awl-shaped tip (Plate XVV, Figures 7 and 8 of [20] and Figure 4E of this study). It is worth noting that both elongated and short awl-shaped tips can be observed from the leaves of one specimen attributed to *Barsassia platyphylla* (Plate XVV, Figure 8 of [20]) and that the specimens attributed to *Barsassia ornata* also show leaves with elongated awl-shaped tip (Figure 4D of this study and Plate XVV, Figures 1 and 9 of [20]). Leaf shape difference is not shown to distinguish *Barsassia platyphylla* from *Barsassia ornata*—the two species of which are identical.

Dou et al. (1983) [9] studied the specimens from the Middle Devonian of Junggar, Xinjiang, China and combined *Barsassia ornata* and *Blasaria sibirica* (Kryshtofovich, 1927) Zalessky, 1934 [35], to *Barsassia sibirica* according to the priority of names (Articles 11–12 of [15]). The plant of new combination is characterized by hexagonal or rectangular leaf base and fan-shaped leaf with distal tip [9]. However, fan- or rectangular-shaped leaves with finger-like tips (Plate 201 Figures 3, 4, 5, 9, 11 and 12 of [9]) and hexagonal leaf base (Plate 201 Figures 1, 6, 7, 8 and 10 of [9]) were not seen from single specimen. Such characters seem not evident or sufficient to combine the two species. It is also worth noting that hexagonal leaf base is not a distinct character and was also reported in other coeval plants, such as lycopsid species of *Gilboaphyton* from the Middle Devonian of Western Venezuela, New York, USA [36] and the Late Devonian of North Xinjiang, China [37] and *Archaeosigillaria* from the Middle Devonian of North Xinjiang [9]. *Blasaria sibirica* is characterized by hexagonal leaf base but no information of leaf morphology [37], whilst *Barsassia ornata*, fan-shaped leaf with leaf tip or rectangular-shaped leaf with finger-like tip. They are two different plants.

Huang (1983) [38] followed the combination of Dou et al. (1983) [2] and reported the species under the name *Barsassia sibirica* from the Middle Devonian of Zhangguangcai Mountains, Heilongjiang Province, northeastern China. The plant of Huang (1983) [38] shows the rectangular-shaped leaf with a finger-like tip and step-like structure, being similar to that shown by our specimens from Xinjiang (Figure 2). The specimens under the name of *Barsassia sibirica* in Huang (1983) (Plate II, Figures 4–6 of [38]) are transferred to *Barsassia ornata*.

Senkenvitsch (1991) [21] established *Barsassia grandis* and *Barsassia plana* based on the materials from the Middle Devonian of Kazakhstan. *Barsassia grandis* is characterized by the rectangular-shaped leaf with a finger-like tip; such character is also seen in specimens of the present study (Figure 2 and Figure 3D–F) and Dou et al. (1983) [9] (Plate 201, Figure 11 of [9] and Table 1, Figure 4F of this study). *Barsassia plana* has the broadly triangular leaf with a short awl-shaped tip and flat leaf surface (Plate LXIV, Figure 6 of [21] and Figure 4I of this study). From our specimens from Xinjiang, the fan-shaped leaf (Figure 1A and Figure 4K) and short awl-shaped leaf tip (Figure 1A and Figure 4M) are both shown broadly. In addition, all these morphological characters are seen from the present specimens, showing a variation of leaf morphology within one species, *Barsassia ornata*.

Snigirevskaya and Bogdanova (1992) [31] studied the specimens from the Middle Devonian of Kuzanets Basin, southwestern Siberia, Russia, and identified epidermal and anatomical characters of *Barsassia ornata* as deeply submerged stomata, star-shaped stele, and spirally thickened xylem tracheids. They assigned *Barsassia ornata* to the Family Asteroxylaceae accordingly. Snigirevskaya and Bogdanova (1992) [31] chose Figure 1 of Zalessky (1933) [17] as the lectotype of *Barsassia ornata*. However, the lectotype is invalid because the type of a fossil-taxon at the rank of species or below is always a specimen (Article 8.5 of [22]).

Snigirevskaya (2010) [34] compared *Barsassia ornata* with *Orestovia devonica* and thought that *Barsassia* belonged to the higher land plant according to the deeply submerged stomata. The specimens attributed to *Barsassia ornata* in Snigirevskaya (2010) [34] are well preserved and show dichotomous branching. The leaf is fan shaped with a short-awl tip, such is similar to the materials from Xinjiang (Figure 1B, Table 1 of this study and Plate 201, Figure 4 of [9]).

The leaf shapes of *Barsassia ornata* are fan shaped or rectangular shaped. The fan shaped is basic leaf shape of *Barsassia ornata* as is commonly seen in the specimens. The angle of the two upper edges of the fan-shaped leaf is nearly 180° to form rectangular-shaped leaves in the surface view. The two leaf shapes might be at different growth stages.

## 5. Conclusions

Fossil plant *Barsassia ornata* (Lycopsida) is studied in terms of morphology and nomenclature based on the materials from the Middle Devonian Hujiersite Formation, Hoboksar County, West Junggar, Xinjiang, China, and the International Code of Nomenclature for algae, fungi, and plants (Shenzhen Code). *Barsassia ornata* is determined as the type species of the genus *Barsassia*, and its neotype is designated from our collection. *Barsassia ornata* is a herbaceous lycopsid consisting of easily recognized and characteristic step-like stem and tight, pseudowhorls, and imbricate fan- or rectangular-shaped leaves. *Barsassia ornata* can be used in dating and correlating the Middle Devonian terrestrial strata. *Barsassia ornata* formed the Devonian coal and is potentially related to petroleum reserve.

## Figures and Tables

**Table 1:** Dimensions and characteristics of *Barsassia ornata* under different names.

Name	Stem				Leaf			Locality	Horizon	References
	Width/mm	Length/mm	Dichotomously Branched	Anatomy	Length/mm	Width of the Base/mm	Leaf Characteristic			
*Barsassia ornata*	3	Unknown	Unknown	Unknown	5	3–11	Isosceles triangular leaf with a slightly pointed tip and the surface of leaf cover with points	Barzas, Russia	D_2_	[17]
*Barsassia platyphylla*	Unknown	Unknown	Unknown	Unknown	8	6	Triangular leaf with a wide base and an elongated awl-shaped tip	Barzas, Russia	D_1-2_	[20]
*Barsassia ornata*	3	10	Unknown	Unknown	10–11	7	Triangular leaf with an expanded base and a narrow awl-shaped tip	Russia	D_2_	[20]
*Barsassia sibirica*	40, mean value = 20	Unknown	Unknown	Unknown	Unknown	Unknown	Triangular leaf with an expanded base	Xinjiang, China	D_2_	[9]
*Barsassia grandis*	1.6–17	3.5–16	Unknown	Unknown	7.5–8	4–17	Rectangular-shaped leaf with a finger-like tip	Katbas Mountains, Kazakhstan	D_2_	[21]
*Barsassia plana*	16	49	Unknown	Unknown	4–16	6–20	Broadly triangular leaf with short-awl-shaped tip, flat leaf surface	Kazakhstan	D_2_	[21]
*Barsassia ornata*	Unknown	30	Unknown	Xylem consisting of spiral and stair tracheids	5	5	Isosceles triangular leaf with an expanded base and a narrow protruding tip	Russia	D_2_	[31]
*Barsassia ornata*	Unknown	Unknown	Yes	Unknown	Unknown	Unknown	Triangular leaf	Russia	D_2_	[34]
*Barsassia ornata*	6.6–12	161	Yes	Unknown	1.7–4.0	3.7–4.5	Fan-shaped leaf or rectangular-shaped leaf	Xinjiang, China	D_2_	This study

## Data Availability

All studied fossil specimens are housed at the Nanjing Institute of Geology and Palaeontology, Chinese Academy of Sciences, with reference numbers and the prefix PB. All related data of this study are given in Table 1 of this paper.

## References

[1] Gensel P.G., Andrews H.N. (1984). Plant Life in the Devonian.

[2] Stein W.E., Berry C.M., Hernick L.A., Mannolini F. (2012). Surprisingly complex community discovered in the mid-Devonian fossil forest at Gilboa. Nature.

[3] Berry C.M., Marshall J.E.A. (2015). Lycopsid forests in the early Late Devonian paleoequatorial zone of Svalbard. Geology.

[4] Berner R.A. (1997). The Rise of Plants and Their Effect on Weathering and Atmospheric CO_2_. Science.

[5] Morris J.L., Leake J.R., Stein W.E., Berry C.M., Marshall J.E.A., Wellman C.H., Milton J.A., Hillier S., Mannolini F., Quirk J. (2015). Investigating Devonian trees as geo-engineers of past climates: Linking palaeosols to palaeobotany and experimental geobiology. Palaeontology.

[6] Xue J.Z., Deng Z.Z., Huang P., Huang K.J., Benton M.J., Cui Y., Wang D.M., Liu J.B., Shen B., Basinger J.F. (2016). Belowground rhizomes in paleosols: The hidden half of an Early Devonian vascular plant. Proc. Natl. Acad. Sci. USA.

[7] Gensel P.G., Edwards D. (2001). Plants Invade the Land.

[8] Petrosyan N.M., Oswald D.H. (1967). Stratigraphic importance of the Devonian flora of the USSR. International Symposium on the Devonian Systems.

[9] Dou Y.W., Sun Z.H., Wu S.Z., Gu D.Y. (1983). Devonian Plants of Xinjiang. Palaeontological Atlas of Northwestern China.

[10] Zeng Y.C., Xiao S.L. (1991). The Palaeozoic Earthem of Xinjiang (No. 2 Stratigraphic Summary of Xinjiang).

[11] Xiao S.L., Hou H.F., Wu S.Z. (1992). The Researches of Devonian System in North Xinjiang.

[12] Xu H.H., Jiang Q., Zhang X.L., Wang Y., Feng J. (2015). On the Mid Devonian Hujiersite flora from West Junggar, Xinjiang, China, its characteristics, age, palaeoenvironment and palaeophytogeographical significances. Acta Palaeontol. Sin..

[13] Liu B.C., Zong R.W., Wang Y., Xu H.H. (2021). On the age of Devonian ancient petroleum reservoir in west Junggar, Xinjiang, Northern Xinjiang, China. J. Stratigr..

[14] Sun D.W., Wang Y., Xu H.H., Fu Q. (2007). Restudy on the cuticles of Late Devonian coal from Luquan, Yunnan, China. Acta Palaeontol. Sin..

[15] Xu H.H., Marshall J.E.A., Wang Y., Zhu H.C., Berry C.M., Wellman C.H. (2014). Devonian spores from an intra-oceanic volcanic arc, West Junggar (Xinjiang, China) and the palaeogeographical significance of the associated fossil plant beds. Rev. Palaeobot. Palynol..

[16] Song D.F., Wang T.G., Zhong N.N., Chen Y., He D.F., Li D. (2021). Discovery of cutinitic liptobiolith in northwestern China and a comparative study with Luquan Devonian coal. Sci. China Earth Sci..

[17] Zalessky M.D. (1933). Observations sur trois vegetaux nouveaux paleozoiques. Acad. Sci. U.R.S.S. Bull..

[18] Gomankov A.V. (2019). *Orestovia*-like plants from the Devonian of Russia: Morphology and taxonnmic position. Lethaea Ross..

[19] Han D.X. (1989). The features of Devonian beds in centural Yunnan, China. Int. J. Coal Geol..

[20] Stepanov S.A. (1975). Phytostratigraphy of Reference Sections Devonian Suburbs of Kuzbass.

[21] Senkevitsch M.A., Dubatolov V.N., Stukalina G.A. (1991). Biostratigraphy of the Lower and Middle Devonian of the Dzungar-Balkhash Province.

[22] Turland N.J., Wiersema J.H., Barrie F.R., Greuter W., Hawksworth D.L., Herendeen P.S., Knapp S., Kusber W.H., Li D.Z., Marhold K. (2018). International Code of Nomenclature for algae, fungi, and plants (Shenzhen Code) adopted by the Nineteenth International Botanical Congress Shenzhen, China, July 2017.

[23] Xu H.H., Wang Y., Berry C.M., Cai C.Y. (2008). Two species of *Haskinsia* Grierson et Banks (Lycopsida) from the Middle Devonian of Xinjiang, China, and consideration of their palaeogeographical significance. Bot. J. Linn. Soc..

[24] Xu H.H., Berry C.M., Wang Y., Marshall J.E.A. (2011). A new species of *Leclercqia* Banks, Bonamo et Grierson (Lycopsida) from the Middle Devonian of North Xinjiang, China, with a possible climbing habit. Int. J. Plant Sci..

[25] Xu H.H., Wang Y. (2011). A neotype for *Colpodexylon gracilentum* Dou (Lycopsida) from the Middle Devonian of North Xinjiang, China. J. Syst. Evol..

[26] Xu H.H., Wang Y., Wang Q. (2012). A new homosporous, arborescent lycopsid from the Middle Devonian of Xinjiang, Northwest China. Palaeontology.

[27] Xu H.H., Feng J., Jiang Q., Wang Y. (2013). Report of *Drepanophycus* Göppert (Lycopsida) from the Middle Devonian of Xinjiang, China. J. Syst. Evol..

[28] Jiang Q., Wang Y., Xu H.H., Feng J. (2013). A new species of *Aneurophyton* (Progymnospermopsida) from the Middle Devonian of West Junggar, Xinjiang, China, and its paleophytogeographical significance. Int. J. Plant Sci..

[29] Zheng D.R., Xu H.H., Wang J., Feng C.Q., Zhang H.C., Chang S.C. (2016). Geochronologic age constraints on the Middle Devonian Hujiersite flora ofXinjiang, NW China. Palaeogeogr. Palaeoclimatol. Palaeoecol..

[30] Fairon-Demaret M., Hilton J., Berry C.M., Jones T.P., Rowe N.P. (1999). Surface preparation of macrofossils (dégagement). Fossil Plants and Spores: Modern Techniques.

[31] Snigirevskaya N.S., Bogdanova L.A. (1992). Finding of stomata and xylem in plants of the genus *Barsassia* (Asteroxylaceae, Lycopodiophyta) from the Middle Devonian of the Kuznets basin and some questions of the stomatographic study of ancient plants. Bot. Zhurnal.

[32] Kidston R., Lang W.H. (1920). On old red sandstone plants showing structure, from the Rhynie Chert bed, Aberdeenshire. Part III. *Asteroxylon Mackiei*, Kidston and Lang. Trans. R. Soc. Edinb..

[33] Taylor T.N., Taylor E.L., Krings M. (2009). Paleobotany:The Biology and Evolution of Fossil Plants.

[34] Snigirevskaya N.S. (2010). Status of lycopsida and some Devonian coal-fromation problems. Bot. Mag..

[35] Zalessky M.D. (1934). On the new Devonian plant *Blasaria sibirica* gen. et sp. nov. Bull. USSR Acad. Sci..

[36] Berry C.M., Edwards D. (1997). A new species of the lycopsid Gilboaphyton Arnold from the Devonian of Venezuela and New York State, with a revision of the closely related genus Archaeosigillaria Kidston. Rev. Palaeobot. Palynol..

[37] Liu B.C., Bai J., Wang Y., Yang N., Xu H.H. (2021). On the discovery of *Gilboaphyton* (Lycopsida) from the Upper Devonian of East Junggar, Xinjiang, and its global distribution. Rev. Palaeobot. Palynol..

[38] Huang B.H. (1983). The Middle Devonian Plants from the Northern Part of Zhangguangcai Mts.

